# Effects of Dietary Limonene Supplementation on Growth Performance and Immunological Parameters of Common Carp, *Cyprinus carpio*, Challenged by *Aeromonas hydrophila*

**DOI:** 10.3390/ani13203197

**Published:** 2023-10-13

**Authors:** Morteza Yousefi, Seyyed Morteza Hoseini, Afaf N. Abdel Rahman, Yury Anatolyevich Vatnikov, Evgeny Vladimirovich Kulikov, Elena Valentinovna Kharlitskaya, Sergey Borisovich Seleznev

**Affiliations:** 1 Department of Veterinary Medicine, RUDN University, Miklukho-Maklaya St., 117198 Moscow, Russia; vatnikov@yandex.ru (Y.A.V.); kulikov-ev@rudn.ru (E.V.K.); kharlitskaya-ev@rudn.ru (E.V.K.); seleznev-sb@rudn.ru (S.B.S.); 2 Inland Waters Aquatics Resources Research Center, Iranian Fisheries Sciences Research Institute, Agricultural Research, Education and Extension Organization, Gorgan 4916687631, Iran; 3 Department of Aquatic Animal Medicine, Faculty of Veterinary Medicine, Zagazig University, Zagazig P.O. Box 44511, Egypt; afne56@gmail.com

**Keywords:** disease, dietary additive, innate immunity, mucus, humoral

## Abstract

**Simple Summary:**

Functional feeds are promising tools to increase disease resistance in fish and prevent the rise of antibiotic-resistant bacteria. Here, we found that dietary 200 mg/kg limonene can improve feed efficiency, modulate blood granulocytes, cause humoral and mucosal innate immunity, and intestinal bacteria composition in common carp, *Cyprinus carpio*, which subsequently mitigated the fish mortality after *Aeromonas hydrophila* challenge.

**Abstract:**

This study examined the impact of dietary limonene treatment on the growth performance, immune response, and disease resistance of common carp, *Cyprinus carpio*. The fish were fed with either a control diet (CTL; no limonene supplementation) or four experimental diets containing 50 (50 L), 100 (100 L), 200 (200 L), and 400 (400 L) mg/kg limonene over a 70-day period, followed by *Aeromonas hydrophila* challenge. The 200 L treatment resulted in a significant decrease in FCR compared to the CTL treatment. The highest post-challenge mortality was associated with the CTL treatment (62.7%), while the 200 L treatment had the lowest mortality (30.7%). Before the challenge, dietary limonene significantly increased humoral and skin mucosal immune parameters compared to the CTL treatment. The highest leukocyte, lymphocyte counts, skin mucosal protease activity, and intestinal lactic acid bacteria were observed in the 200 L treatment before the challenge. The highest plasma lysozyme activity was observed in the 400 L treatment, whereas the highest skin mucosal lysozyme and peroxidase activities were observed in the 100 L and 200 L treatments before the challenge. There were no significant differences in the blood neutrophil, monocyte, and eosinophil counts, humoral alternative complement activity, skin mucosal alkaline phosphatase activity, and the intestinal total viable bacteria among the treatments before the challenge. After the challenge, the 200 L treatment exhibited the highest leukocyte, neutrophil, and monocyte count, skin mucosal immune parameters, and intestinal lactic acid bacteria, whereas the highest blood eosinophil count was observed in the 100 L, 200 L, and 400 L treatments. At this time, the lowest blood lymphocyte counts were observed in the 100 L and 200 L, but the lowest intestinal total viable bacteria were observed in the 100 L, 200 L, and 400 L treatments. Based on these findings, dietary limonene at 200 mg/kg is ideal for common carp to promote feed efficiency, innate immunity boosting, and resistance against *A. hydrophila*.

## 1. Introduction

Aquaculture has emerged as a crucial source of global food production, with a record high of 122.6 million tons produced in 2020, valued at USD 281.5 billion. This trend is expected to continue, with an estimated growth of 14 percent in aquatic animal production by 2030 [1]. Common carp, *Cyprinus carpio*, is a highly valued freshwater fish species widely cultured for its economic significance. Global production of common carp is approximately 4.6 million tons, with China, India, Iran, Indonesia, and Bangladesh being the top producers [2]. Carp can be cultivated through various methods, such as ponds, cages, and tanks, and is an affordable and nutritious protein source for human consumption, making it a significant food fish worldwide [3].

In order to ensure the long-term viability of the aquaculture industry and maximize profitability, sustainable growth is crucial. However, disease outbreaks pose a significant challenge, potentially leading to economic losses and environmental impacts [4]. One of the pathogens causing serious disease outbreaks in fish and shellfish is *Aeromonas hydrophila*, a bacterium responsible for *Aeromonas* septicemia [5]. The bacterium can infect fish through wounds, ingestion, or the gills, causing severe tissue damage, hemorrhaging, and even death. The overuse of antibiotics to treat *Aeromonas* septicemia in fish led to the rise of antibiotic-resistance strains; therefore, while various antibiotics are currently used to treat this condition, it is crucial to avoid their overuse [6,7]. Promoting good management practices that improve the innate immune system and prevent the occurrence of diseases of fish is crucial.

Feed is a critical component of fish farming as it provides the necessary nutrients for fish to maintain their health and growth [8]. In aquaculture, functional feed refers to specialized fortified feeds that offer additional health benefits beyond basic nutrition [9]. These additives include immunostimulants, probiotics, prebiotics, and enzymes, among others. Incorporating functional feeds in aquaculture practices can help farmers improve fish growth, disease resistance, and overall health while reducing the use of antibiotics and other chemical treatments [10]. Herbal additives are becoming increasingly popular in aquaculture as a natural alternative to synthetic chemicals. These additives, derived from medicinal plants, have been shown to possess antimicrobial and immunostimulatory properties that can enhance fish health and growth [11]. Limonene is a cyclic monoterpene found mainly in the peel oil of oranges, grapefruit, and lemons [12]. It exhibits potent antimicrobial, antioxidant, anti-inflammation, gastroprotective, and anxiolytic properties in vitro and in vivo [13,14,15,16,17], which makes it a versatile bioactive compound. In addition, dietary supplementation of limonene has been found to improve the growth performance of Nile tilapia, *Oreochromis niloticus*. This effect is thought to be associated with an increase in the intestinal absorptive area and modulation of gene expression related to metabolism and somatic growth [18]. Furthermore, research has found that limonene can also enhance immune strength and resistance against *Yersinia ruckeri* in rainbow trout, *Oncorhynchus mykiss*, as a feed additive [19]. However, no studies have been conducted on the effects of dietary limonene on other fish species and photogenic bacteria. Therefore, the present study aims to evaluate the impact of dietary limonene supplementation on growth performance, immunological responses, and resistance against *A. hydrophila* infection in common carp.

## 2. Materials and Methods

### 2.1. Ethical Statement

This study was approved by the Bioethics Commission of Agrarian and Technological Institute, RUDN University (Protocol № 11 dated 14 November 2022).

### 2.2. Diets and Fish Maintenance

The diets were formulated using WUFFDA, according to the nutritional requirements of common carp [20]. A basal diet (CTL) was prepared using locally-sourced feedstuffs, as shown in Table 1. Four experimental diets were formulated by adding 50 (50 L), 100 (100 L), 200 (200 L), and 400 (400 L) mg/kg limonene (Sigmaalderich, Saint Louis, MO, USA) to the basal diet, replacing cellulose. These concentrations were chosen based on studies on other fish species, showing 400 mg/kg limonene improves fish growth and intestinal morphology [21]. The feedstuffs were finely ground, mixed for 30 min, moisturized with 400 mL/kg water, and pelleted using a meat grinder. The resulting pellets were dried using a fan blower and stored in a refrigerator until use.

For this experiment, common carp juveniles that weighed approximately 22 g per individual were used. Four hundred fish were stocked in 15 plastic tanks (30 individuals per tank). The tanks were filled with 150 L of water and were equipped with aeration and a water flow rate of 0.5 L/min. To ensure the fish were acclimatized to the experimental conditions, they were fed the CTL diet for a week. After that, the tanks were divided into five treatments (three replications), where the fish were fed either the CTL, 50 L, 100 L, 200 L, or 400 L diets for 70 days. The daily feeding rate was 2.5% of biomass divided into three meals, which was adjusted every other week based on the tanks’ biomasses.

### 2.3. Growth Performance

Growth performance and feed efficiency were calculated at the end of the 70-day feeding period using the following formulae:

$$\mathrm{Specific}\;\mathrm{growth}\;\mathrm{rate}\;\left(\mathrm{SGR};\;\%/d\right)=100\times \frac{\mathrm{Ln}\;\left(\mathrm{final}\;\mathrm{weight}\right)-\mathrm{Ln}\;\left(\mathrm{initial}\;\mathrm{weight}\right)}{t}$$

$$\mathrm{Feed}\;\mathrm{conversion}\;\mathrm{ratio}\;\left(\mathrm{FCR}\right)=\frac{\mathrm{Consumed}\;\mathrm{feed}\;\left(g\right)}{\mathrm{Gained}\;\mathrm{biomass}\;\left(g\right)}$$

$$\mathrm{Weight}\;\mathrm{gain}\;\left(\%\right)=100\times \frac{\mathrm{final}\;\mathrm{weight}\;\left(g\right)-\mathrm{initial}\;\mathrm{weight}\;\left(g\right)}{\mathrm{Initial}\;\mathrm{weight}\;\left(g\right)}$$

as “t” was the duration of fish rearing (i.e., 70 days).

### 2.4. Bacterial Challenge 

*A. hydrophila* was isolated from moribund fish (presenting the signs of *Aeromonas* septicemia) and confirmed by the colony appearance (white to grey, semi-transparent, and convex colonies), motility, and biochemical examinations (gram-negative and catalase-, oxidase-positive). The final colonies were tested by PCR using a primer of lipase gene, designed before [22]. The bacterium was cultured overnight on tryptic soy broth. The resulting suspension was then centrifuged for 15 min at 3000 rpm, washed thrice with phosphate buffer (0.1 M; pH 7.2), and re-suspended in the same buffer. The cell density was adjusted to 10^8^ cell/mL (by preparing 0.5 McFarland standard at 625 nm and further 1.5-fold dilution with the buffer), and then each fish was intraperitoneally injected 0.2 mL of the suspension. Prior to the injection, the fish were anesthetized individually. The post-infection mortality was removed three times daily until 14 days.

### 2.5. Sampling, Processing and Analysis

The experiment involved sampling the fish at the end of the feeding period (before the challenge) and 3 days after the bacterial challenge [23,24]. At each time, 9 fish were sampled from each treatment. Blood was then sampled from the caudal vein using heparinized syringes. The fish were then placed in a plastic bag containing 5 mL of buffer (50 mM Tris-HCl, 150 mM NaCl, pH 8.0) and gently rubbed for 1 min to collect the skin mucus. Finally, the fish were dissected by spinal cord dissection, and a piece of the posterior intestine was sampled. 

The blood samples were divided into two portions, which were used for the leukocyte (WBC) counting and plasma separation. WBCs were counted using a Neubauer chamber, and a WBC differential count was performed after the preparation of blood smears stained with Giemsa [25]. 

The blood plasma was separated through centrifugation at 4000× *g* for 7 min at 4 °C and then stored at −70 °C until analysis. In order to determine the plasma lysozyme activity, a turbidimetric method was utilized, as described by Ellis [26]. This method involved using *Micrococcus lysodeikticus*, which was suspended in phosphate buffer (pH of 6.2). The suspension was mixed with the sample, and the optical density was monitored at 500 nm for 5 min. Each 0.001 decrease in absorbance per min was considered as one unit of lysozyme activity. The alternative complement (AC) activity was determined based on the hemolytic activity of the plasma samples against sheep RBC. In order to do this, the plasma samples were serially diluted (ranging from 0.156% to 5%) in gelatin-vernal buffer containing magnesium and EGTA, mixed with sheep RBC, and then incubated at room temperature for 90 min. The reaction was stopped by adding EDTA, and the hemolysis rate was determined by a spectrophotometer. Finally, the AC activity was calculated based on the method described by Yano [27].

The collected skin mucus was poured from the plastic bags into a tube and centrifuged (13,000× *g*; 20 min; 4 °C), and the supernatant was collected and kept at −70 °C until analysis. The soluble protein concentration of the supernatants was determined based on Bradford [28]. The mucosal lysozyme activity was determined as mentioned above. The mucosal alkaline phosphatase (ALP) activity was determined using a commercial kit (Dialab Co., Grozny, Russia). The mucosal peroxidase activity was determined based on 3,3′,5,5′-tetramethylbenzidine hydrochloride reaction with hydrogen peroxide, as described by Guardiola et al. [29]. The mucosal protease activity was determined based on the reaction with azocasein (4 °C; 19 h), as suggested by Guardiola, Cuesta, Arizcun, Meseguer and Esteban [29].

The intestinal samples were homogenized in 10 volumes of 0.85% sodium chloride solution. The homogenates were then serially diluted 10^1^–10^5^ times, and 0.1 mL of each dilution was cultured on both nutrient agar and MRS agar to determine total viable bacteria (TVB) and lactic acid bacteria (LAB) populations, respectively. The plates (10 cm in diameter) were incubated at room temperature for 72 h. Then, the colony-forming units were counted, and cell numbers per g fish intestine were calculated. 

### 2.6. Statistical Analysis

The study followed a completely randomized design, using each fish tank as a replication. Statistical analysis was performed using the mean of sub-samples from each tank. The normality of data was confirmed using the Shapiro-Wilk test, while variance homogeneity was confirmed using the Levene test. One-way ANOVA was used to analyze growth and post-challenge mortality data, while two-way repeated measure ANOVA was used for other parameters (dietary limonene levels × sampling time). In order to identify significant differences among treatments, the Duncan test was conducted, with a significance level set at *p* < 0.05. Mean ± pooled standard error was used to present data. All analyses were performed using SPSS v.22.

## 3. Results

### 3.1. Growth Performance

Table 2 presents growth performance and feed efficiency data. No significant differences were observed in final weight, SGR, and weight gain among treatments. However, FCR in the 200 L treatment was significantly lower than in the other treatments. 

### 3.2. Post-Challenge Mortality

Post-challenge mortality began 3 days after infection in the CTL, 50 L, and 100 L treatments. However, in the 200 L and 400 L treatments, the first mortality was observed 5 and 4 days after infection, respectively. Mortality was stopped in all treatments after 11 days. At the end of the challenge period, the highest mortality was observed in the CTL treatment (62.7%), significantly higher than in the other treatments. The 200 L treatment had the lowest mortality (30.7%), significantly lower than in the other treatments. The mortality rates in the 50 L (48%), 100 L (44%), and 400 L (48%) treatments were statistically similar (Table 3).

### 3.3. Blood Leukocytes

Table 4 presents blood leukocyte counts. Before the challenge, the 200 L treatment showed significantly higher WBCs, compared to the CTL treatment. At this time, the 200 L and 400 L treatments had significantly higher lymphocyte counts than the CTL treatment. However, there were no significant changes in the blood neutrophil, monocyte, and eosinophil counts among the treatments before the challenge. The bacterial challenge significantly increased WBC, neutrophil, monocyte, and eosinophil counts in all treatments. At this time, lymphocyte count significantly decreased in the 100 L, 200 L, and 400 L treatments compared to before the challenge. The highest WBC, neutrophil, and monocyte counts after the challenge were observed in the 200 L treatment. The highest post-challenge eosinophil count was observed in the 100 L, 200 L, and 400 L treatments, whereas the lowest lymphocyte count was observed in the 100 L and 200 L treatments.

### 3.4. Plasma and Mucosal Innate Immune Parameters

Table 5 shows that bacterial challenge significantly increased plasma lysozyme, mucosal lysozyme, and mucosal peroxidase activities in all treatments. The CTL treatment had the lowest plasma lysozyme activity, whereas the 200 L treatment had the highest activity before and after the challenge. Mucosal lysozyme and peroxidase activities significantly increased in the 100 L and 200 L treatments before the challenge, compared to the CTL treatment. After the challenge, the CTL treatment exhibited significantly lower mucosal lysozyme and peroxidase activities when compared to the other treatments. The 200 L treatment had significantly higher mucosal lysozyme activity before and after the challenge compared to the other treatments. 

Plasma AC activity did not show significant differences among treatments before the challenge. After the challenge, the plasma AC activity increased significantly in the 50 L, 100 L, and 200 L treatments compared to the CTL treatment. Dietary limonene and bacterial challenge had no significant effects on mucosal ALP activity. Mucosal protease activity in the 200 L treatment significantly increased compared to the CTL treatment before the challenge. After the challenge, the 100 L and 200 L treatments showed significantly higher mucosal activities compared to the CTL treatment (Table 5). 

### 3.5. Intestinal Bacteria

Before the challenge, limonene did not induce any changes in the intestinal TVB population. However, after the challenge, the intestinal TVB population significantly increased in all treatments. An increase in dietary limonene level significantly decreased the post-challenge intestinal TVB population in a dose-dependent manner (Table 6). The bacterial challenge significantly decreased the intestinal LAB population in all treatments. Nevertheless, the 100 L and 200 L treatments had significantly higher intestinal LAB populations, before and after the challenge, and the highest population was observed in the 200 L treatment (Table 6).

## 4. Discussion

Limonene has been found to promote fish growth performance and feed efficiency through various mechanisms. Studies have shown that dietary supplementation with pure limonene or limonene-rich additives significantly increases the growth rate of Nile tilapia, along with elevations in the intestinal absorptive surface [18,21,30], gene expression of certain nutrient-transporting molecules [18], and hepatic protein expression related to lipid/carbohydrate metabolism [18,31] and somatic growth [18,30]. In the present study, it was observed that limonene at 200 mg/kg can increase feed efficiency in common carp, likely through these mechanisms, which can improve the economic profitability of carp rearing. However, the optimum dietary limonene in this study was half of that reported for Nile tilapia [18], and further evaluations are needed to determine the underlying mechanisms. 

The non-specific immune system of fish plays a crucial role in preventing pathogens from invading and colonizing their bodies [32]. Therefore, enhancing the non-specific immune system is a reliable strategy to prevent disease outbreaks in aquaculture. This system is composed of several components, including humoral and mucosal elements [33]. Leukocytes play vital roles in the immune defense by carrying out various functions such as phagocytosis, antibody production, inflammation, and secretion of germ-killing molecules [34]. In fish, a common response to infection is an increase in the number of leukocytes, especially granulocytes [35]. Among granulocytes, neutrophils are a class of phagocytic cells that produce bactericidal agents and superoxide anions, making them a crucial element in the fish immune defense system [36]. Monocytes play various immune roles, such as phagocytosis, cytokine production, and antigen presentation. They have the ability to migrate to different tissues and transform into macrophages, which exhibit strong antibacterial and phagocytic activities [37]. Eosinophils, though rare, are crucial in inflammatory responses during infections. Granulocytes are involved in rapid, non-specific immune reactions, leading to an increase in their population shortly after infection [38]. Meanwhile, lymphocytes primarily produce antibodies against antigens, but this response is slower and typically takes weeks to develop after infection [38]. Based on the above information, it can be inferred that granulocytes play a crucial role in the rapid elimination of pathogens. Studies have shown that silver catfish, *Rhamdia quelen*, injected with *A. hydrophila,* had elevated WBC and granulocyte populations 10 days later [39], while common carp infected with the same pathogen showed similar results after 7 days [35]. However, there are variations in immune responses depending on factors such as fish species, pathogen type, and the time of sampling. For instance, Nile tilapia exhibited an increase in WBC and lymphocyte counts but a decrease in monocyte count 24 h after infection with *Enterococcus* sp. [40]. Variations in immune responses may depend on a range of factors, including the time of sampling, the type of pathogen, and the species of fish under investigation. The present study found that the common carp exhibited higher survival rates after being exposed to *A. hydrophila* infection when they had higher levels of neutrophils, monocytes, and eosinophils before and/or after the challenge. These findings suggest that limonene may enhance the fish’s resistance against infection by increasing the number of leukocytes that respond quickly to the pathogen.

Humoral immunological parameters provide reliable indicators of the immune system activation following bacterial challenges. Lysozyme, a bactericidal agent, increases in response to bacterial challenges, effectively counteracting the presence of pathogens in the fish body and promoting their elimination. This enzyme also stimulates phagocytosis of granulocytes, which is pivotal for early pathogen elimination [41]. The present findings are supported by studies observing elevations in humoral lysozyme activity in fish after experimental infection with *A. hydrophila* [23,42,43,44,45,46]. Additionally, the present study found that dietary treatment with limonene, particularly at the 200 mg/kg level, resulted in an increase in lysozyme activity both before and after the bacterial challenge. This suggests that limonene may play a role in inducing lysozyme and enhancing disease resistance in fish. Although research on this topic is limited, a study conducted on rainbow trout showed that dietary limonene (derived from orange peel) administered at 1 and 3 mL/kg significantly increased survival rates after infection with *Y. ruckeri*. However, no significant changes in humoral lysozyme activity were observed [19]. Similarly, a study on Nile tilapia reported an increase in humoral lysozyme activity after supplementation with limonene-rich essential oils (93.76% and 91.45%), but no data on disease resistance were provided [30].

The complement system plays a vital role in the immune system. Complement proteins are involved in various functions, such as cell lysis, opsonization, and inflammation, in the innate immune system. Moreover, the complement system is involved in adaptive immune responses by linking the innate and adaptive arms of immunity; they stimulate B cells to produce antibodies [47]. The function of complement proteins is especially crucial in lysozyme activity against gram-negative bacteria, as they break the outer layer of bacteria, facilitating the access of lysozyme to the bacteria peptidoglycan layer [41]. In the current study, it was observed that there was no change in AC activity before the challenge after the administration of limonene. Although there is no comparable study available for comparison, previous research has shown that the dietary supplementation of a limonene-containing commercial product [Digestrom P.E.P; Defaee et al. [48]] or limonene-containing peel [*Citrus limon*; Chekani et al. [49]] did not result in any change in humoral AC activity in beluga, *Huso huso*, and rainbow trout, respectively. Activation of the complement system occurs during bacterial diseases, including *A. hydrophila* infection [45,46,50,51]. However, gram-negative bacteria similar to *A. hydrophila* can deactivate complement proteins through various means [52]. Following infection with gram-negative bacteria, there is a time-dependent reduction in AC activity, with an initial decline followed by recovery after 3–4 days, as noted by Ravindra et al. [53] and Mohanty and Sahoo [54], 3–4 days after infection with *Flavobacterium columnare* and *Edwardsiella tarda*, respectively. The AC results obtained in the CTL treatment after the challenge are consistent with these studies. Moreover, it was observed that the fish treated with limonene, especially those treated with L200, experienced AC elevations after the challenge. This suggests that limonene may have prevented complement deactivation caused by *A. hydrophila* and/or decreased the bacterium population, which ultimately led to less complement protein deactivation, as partially supported by the results of the intestinal bacterial population analysis.

The skin mucus plays a crucial role in protecting fish against waterborne pathogens by containing several immune-related components [55]. Lysozyme activity has been found in fish skin mucus, acting as a bactericidal agent similar to humoral lysozyme. Protease activity of the skin mucus can hamper its integrity, leading to the constant washing out of foreign germs [56]. Additionally, peroxidase activity participates in forming peroxidase-H_2_O_2_-halide, which is a potent bactericidal complex [56]. Considering the elevations in the skin mucosal lysozyme, peroxidase, and protease activities in the present study, dietary limonene was able to improve mucosal immunity, particularly in the 200 L treatment. This is especially important in fish farms, where skin immunity plays a crucial role in preventing pathogen infiltration during disease outbreaks. While information about the role of limonene in skin mucosal immunity is scarce, the present results are similar to those obtained using other phytochemicals in common carp. For example, *Myristica fragrans* extract [57], *Origanum majorana* extract [58], and oak acorn + coriander + common mallow extract [59] have all demonstrated improvement in the skin mucosal immunity and resistance against *A. hydrophila* infection. Intraperitoneal injection of bacteria results in systemic infections in fish [24] that activate the immune responses in all organs of the fish. Common carp in the present study exhibited such responses after the challenge with *A. hydrophila,* and the limonene-treated fish (particularly the 200 L treatment) expressed higher mucosal immune responses that can be beneficial in protecting against waterborne pathogens.

The intestinal microbial communities have important roles in fish health, disease resistance, nutrient digestion, and absorption. Environmental, nutritional, and genetic factors affect the intestinal microflora [60]. LABs are one of the important and most-studied bacterial groups in the fish intestine that improve fish growth and disease resistance when they dominate the intestinal microflora [61]. No study has been conducted to demonstrate the roles of dietary limonene on the intestinal microflora in fish, and there are a few studies regarding the importance of dietary herbal additives on fish intestinal microflora. Dietary supplementation with Chinese yam peel has increased the relative abundance of beneficial bacteria, such as *Lactobacillus* sp. and *Bacteriodes* sp., but decreased the relative abundance of harmful bacteria, such as *Pseudomonas* sp. and *Vibrio* sp. in common carp intestine [62]. Dietary thymol or carvacrol supplementation has significantly decreased total anaerobic bacteria in rainbow trout; also, thymol decreased *Lactobacillus* sp. population [63]. Dietary supplementation with *Artemisia annua* extract has significantly increased the *Lactobacillus* sp. and *Enterococcus* sp. population in the intestine of largemouth bass *Micropterus salmoides* [64]. Opportunistic bacteria are present in the environment and fish bodies. Successful colonization is required for inducing diseases from these bacteria. Experimental bacterial challenges have shown such colonizations, particularly in moribund fish [65]. The present results also showed that infection with *A. hydrophila* increased the intestinal TVB, which is believed to be due to the injection of a high concentration of the bacterium into the fish. However, a decrease in the intestinal TVB in the limonene-treated fish suggests the antibacterial effects of this terpenoid. According to da Silva, Bandeira Junior, Cargnelutti, Santos, Gündel, and Baldisserotto [13] and Zhong, Chen, Yang, Tang, Jiang, Guo, and Gao [14], limonene has antibacterial effects against *A. hydrophila*, in vitro, which justify the present results. It is also speculated that the decrease in *A. hydrophila* population may provide space for beneficial bacteria, such as LAB, to colonize in the intestine and excrete their positive effects. 

## 5. Conclusions

Overall, the present research suggests that limonene is a promising supplement for common carp as it can enhance feed efficiency and fish immune system. It can also possibly protect them against waterborne pathogens. Limonene triggered several early-responding components before and after bacterial infection, which are believed to be responsible for the highest survival rates of common carp after the infection. Based on the results, it is hypothesized that limonene increases the resistance of common carp against *A. hydrophila* infection via; 1, an increase in the blood granulocyte count before the infection, followed by a further sharp surge after infection; 2, boosting the systemic bactericidal capacity by increasing plasma lysozyme before and after the infection; 3, preventing decrements in the complement system after infection; 4, combating the entry of pathogens (those potentially leaked form the moribund fish) through the fish surface by surging the skin mucosal immune defense; and 5, increasing the population of LAB in the fish intestine, which has beneficial effects in disease resistance and may compete with pathogens for colonization. The recommended dosage of 200 mg/kg limonene is a useful guideline for fish farmers to follow.

## Figures and Tables

**Table 1:** Ingredients (g/kg) and chemical composition of the diets.

	Diets										
Ingredients	CTL	50 L	100 L	200 L	400 L	Ingredients	CTL	50 L	100 L	200 L	400 L
Corn meal ^1^	100	100	100	100	100	Lysine ^8^	2	2	2	2	2
Wheat meal ^2^	279	279	279	279	279	Limonene ^9^	0	0.05	0.10	0.20	0.40
Soybean meal ^3^	200	200	200	200	200	Cellulose ^10^	1	0.95	0.90	0.80	0.60
Poultry byproduct ^4^	380	380	380	380	380	Proximate composition (g/kg)					
Corn oil	10	10	10	10	10	Moisture	88.5	87.7	87.0	87.1	88.2
Sunflower oil	10	10	10	10	10	Crude protein	384	381	383	380	382
Mineral premix ^5^	10	10	10	10	10	Crude fat	106	107	106	107	110
Vitamin premix ^6^	5	5	5	5	5	Crude ash	53.7	54.2	53.5	54.4	53.8
Methionine ^7^	3	3	3	3	3	Crude fiber	43.4	43.9	43.0	43.7	42.9

^1^ Crude protein 8.80%; crude fat 3.80%; crude ash 1.30%; crude fiber 2.20%; ^2^ Crude protein 15.2%; crude fat 3.10%; crude ash 1.27%; crude fiber 7.62%; ^3^ Crude protein 44.3%; crude fat 1.88%; crude ash 5.32%; crude fiber 3.68%; ^4^ Crude protein 63%; crude fat 16%; crude ash 6%; crude fiber 4%; ^5^ Amineh Gostar Co. (Tehran, Iran). The premix provided the following amounts of vitamin to the diets (per kg): A: 1600 IU; D3: 500 IU; E: 20 mg; K: 24 mg; B3: 12 mg; B5: 40 mg; B2: 10 mg; B6: 5 mg; B1: 4 mg; H: 0.2 mg; B9: 2 mg; B12: 0.01 mg; C: 60 mg; Inositol: 50 mg; ^6^ Amineh Gostar Co. (Tehran, Iran). The premix provided the following amounts of minerals to the diets (per kg): Se: 0.15 mg; Fe: 2.5 mg; Co: 0.04 mg; Mn: 5 mg; Iodate: 0.05 mg; Cu: 0.5 mg; Zn: 6 mg; Choline: 150 mg; ^7^ CheilJedang Co., Seul, Republic of Korea; ^8^ CheilJedang Co., Seul, Republic of Korea; ^9^ Sigmaalderich Co. Saint Louis, MO, USA; ^10^ Sigmaalderich Co. Saint Louis, MO, USA.

**Table 2:** Growth performance and feed efficiency of the fish-fed diets containing different levels of limonene over 70 days. Different letters within a row show significant differences among the treatments (*n* = 3; Duncan).

						Pooled SE	One-Way ANOVA	
	CTL	50 L	100 L	200 L	400 L		*F*	*p*
Initial weight (g)	25.10	25.03	25.03	25.07	25.07	0.02	0.50	0.737
Final weight (g)	71.67	71.00	72.67	74.33	71.00	0.45	3.29	0.057
FCR	1.52 ^b^	1.52 ^b^	1.49 ^b^	1.36 ^a^	1.49 ^b^	0.02	4.19	0.030
SGR (%/d)	1.50	1.49	1.52	1.55	1.49	0.01	3.17	0.063
Weight gain (%)	185.51	183.63	190.28	196.55	183.25	1.79	3.19	0.062
Survival (%)	100	100	100	100	100	0	0	1.000

**Table 3:** Mortality of the fish-fed diets containing different levels of limonene over 70 days and challenged with *A. hydrophila* over 14 days. Significant differences among the treatments within each day are shown by different letters within each row.

						Pooled SE	One-Way ANOVA	
	CTL	50 L	100 L	200 L	400 L		*F*	*p*
3	12.0 ^b^	12.0 ^b^	4.00 ^a^	0.00 ^a^	0.00 ^a^	1.60	11.5	0.001
5	24.0 ^c^	18.7 ^bc^	13.3 ^ab^	5.33 ^a^	8.00 ^a^	2.06	9.11	0.002
7	41.3 ^c^	33.3 ^b^	21.3 ^a^	17.3 ^a^	18.7 ^a^	2.58	38.6	<0.001
9	56.0 ^c^	40.0 ^b^	32.0 ^b^	22.7 ^a^	33.3 ^b^	3.10	25.4	<0.001
11	62.7 ^c^	48.0 ^b^	44.0 ^b^	30.7 ^a^	48.0 ^b^	2.96	14.1	<0.001

**Table 4:** Blood WBC and differential leukocyte count of the fish-fed diets containing different levels of limonene over 70 days and challenged with *A. hydrophila*. Significant differences among the treatment combinations (limonene × challenge) were indicated by different superscript letters in front of the values (*n* = 3; Duncan). BCH: before the bacterial challenge; ACH: 3 days after the bacterial challenge; L: dietary limonene; CH: bacterial challenge.

							Pooled SE	Two-Way ANOVA		
		CTL	50 L	100 L	200 L	400 L		L	CH	L × CH
WBC (10^3^ cell/µL)	BCH	8.87 ^a^	9.52 ^a^	10.60 ^ab^	12.23 ^b^	10.70 ^ab^	0.33	<0.001	<0.001	0.013
	ACH	19.90 ^c^	23.10 ^d^	26.30 ^e^	28.50 ^f^	24.60 ^de^	0.86			
Lymphocyte (10^3^ cell/µL)	BCH	7.57 ^cd^	7.92 ^cde^	8.25 ^def^	9.38 ^f^	8.99 ^ef^	0.20	0.009	<0.001	<0.001
	ACH	6.98 ^bc^	7.02 ^bc^	4.44 ^a^	3.71 ^a^	6.18 ^b^	0.40			
Neutrophil (10^3^ cell/µL)	BCH	0.83 ^a^	0.99 ^a^	1.49 ^a^	1.87 ^a^	1.07 ^a^	0.11	<0.001	<0.001	<0.001
	ACH	9.34 ^b^	11.61 ^c^	16.17 ^e^	18.30 ^f^	13.33 ^d^	0.90			
Monocyte (10^3^ cell/µL)	BCH	0.47 ^a^	0.60 ^a^	0.81 ^a^	0.98 ^a^	0.64 ^a^	0.54	<0.001	<0.001	0.001
	ACH	2.79 ^b^	3.39 ^c^	4.37 ^d^	5.05 ^e^	3.68 ^c^	0.23			
Eosinophil (10^3^ cell/µL)	BCH	0.03 ^a^	0.06 ^a^	0.04 ^a^	0.08 ^a^	0.11 ^a^	0.18	0.104	<0.001	0.207
	ACH	0.79 ^b^	1.08 ^bc^	1.32 ^c^	1.44 ^c^	1.41 ^c^	0.98			

**Table 5:** Plasma and skin mucosal immunological parameters of the fish-fed diets containing different levels of limonene over 70 days and challenged with *A. hydrophila*. Significant differences among the treatment combinations (limonene × challenge) were indicated by different superscript letters in front of the values (*n* = 3; Duncan). BCH: before the bacterial challenge; ACH: 3 days after the bacterial challenge; L: dietary limonene; CH: bacterial challenge.

							Pooled SE	Two-Way ANOVA		
		CTL	50 L	100 L	200 L	400 L		L	CH	L × CH
Plasma lysozyme (U/mL)	BCH	23.67 ^a^	31.00 ^b^	35.67 ^bc^	40.00 ^c^	45.67 ^de^	2.10	<0.001	<0.001	<0.001
	ACH	52.67 ^e^	67.00 ^f^	74.33 ^g^	86.50 ^h^	66.00 ^f^	3.18			
Plasma AC (U/mL)	BCH	114.00 ^ab^	122.67 ^ab^	120.33 ^ab^	125.67 ^ab^	121.00 ^ab^	1.91	<0.001	0.018	0.006
	ACH	108.00 ^a^	130.80 ^bc^	144.30 ^cd^	152.90 ^d^	111.40 ^a^	5.28			
Mucosal lysozyme (U/mg pr.)	BCH	31.00 ^a^	35.33 ^a^	45.00 ^b^	50.33 ^bc^	36.67 ^a^	2.00	<0.001	<0.001	0.288
	ACH	47.67 ^b^	56.33 ^c^	67.33 ^d^	79.33 ^e^	58.00 ^c^	3.15			
Mucosal ALP (U/mg pr.)	BCH	118.00	120.67	118.67	120.67	118.33	1.82	0.992	0.535	0.993
	ACH	120.33	121.33	121.00	120.33	121.33	1.18			
Mucosal peroxidase (U/mg pr.)	BCH	4.43 ^a^	5.13 ^abc^	5.47 ^bc^	5.40 ^bc^	4.57 ^a^	0.15	<0.001	<0.001	<0.001
	ACH	4.83 ^ab^	5.60 ^c^	6.50 ^d^	8.77 ^e^	5.80 ^c^	0.37			
Mucosal protease (U/mg pr.)	BCH	40.23 ^a^	45.40 ^abc^	44.23 ^ab^	50.20 ^cd^	44.17 ^ab^	1.11	<0.001	0.003	0.021
	ACH	43.20 ^ab^	47.47 ^bc^	52.53 ^de^	56.80 ^e^	41.33 ^a^	0.37			

**Table 6:** Intestinal total viable bacteria (TVB) and lactic acid bacteria (LAB) of the fish-fed diets containing different levels of limonene over 70 days and challenged with *A. hydrophila*. Significant differences among the treatment combinations (limonene × challenge) were indicated by different superscript letters in front of the values (*n* = 3; Duncan). BCH: before the bacterial challenge; ACH: 3 days after the bacterial challenge; L: dietary limonene; CH: bacterial challenge.

							Pooled SE	Two-Way ANOVA		
		CTL	50 L	100 L	200 L	400 L		L	CH	L × CH
TVB (10^4^ cell/g intestine)	BCH	169.63 ^a^	170.74 ^a^	168.89 ^a^	171.48 ^a^	159.63 ^a^	1.59	<0.001	<0.001	<0.001
	ACH	507.78 ^c^	492.59 ^c^	437.04 ^b^	433.33 ^b^	366.67 ^b^	13.87			
LAB (10^4^ cell/g intestine)	BCH	1.96 ^d^	2.11 ^de^	2.30 ^e^	2.85 ^f^	1.93 ^d^	0.10	<0.001	<0.001	0.332
	ACH	0.81 ^a^	0.90 ^ab^	1.10 ^b^	1.59 ^c^	0.96 ^ab^	0.08			

## Data Availability

Data are available upon reasonable request from the corresponding authors.
